# Comparative Evaluation of Cu(acac)_2_ and {[Cu(μ-*O*,*O*′-NO_3_) (L-arg) (2,2′-bpy)]·NO_3_}_n_ as Potential Precursors of Electroless Metallization of Laser-Activated Polymer Materials

**DOI:** 10.3390/ma14040978

**Published:** 2021-02-19

**Authors:** Bartłomiej Jagodziński, Piotr Rytlewski, Krzysztof Moraczewski

**Affiliations:** Department of Materials Engineering, Kazimierz Wielki University, 85-064 Bydgoszcz, Poland; prytlewski@ukw.edu.pl (P.R.); kmm@ukw.edu.pl (K.M.)

**Keywords:** infrared lasers, surface activation, electroless metallization, organometallic complex

## Abstract

This paper presents a comparative assessment of Cu(acac)_2_ and {[Cu(μ-*O,O*′-NO_3_) (L-arg)(2,2′-bpy)]·NO_3_}_n_ as potential precursors for the electroless metallization of laser activated polymer materials. Coatings consisting of polyurethane resin, one of the two mentioned precursor compounds, and antimony oxide (Sb_2_O_3_), as a compound strongly absorbing infrared radiation, were applied on the polycarbonate substrate. The coatings were activated with infrared Nd: YAG laser radiation (λ = 1064 nm) and electroless metallized. It was found that after laser irradiation, a micro-rough surface structure of the coatings was formed, on which copper was present in various oxidation states, as well as in its metallic form. For selected parameters of laser irradiation, it was possible to deposit a copper layer on the coating containing Cu(acac)_2_ and Sb_2_O_3_, which is characterized by high adhesion strength. It was also found that the {[Cu(μ-*O,O*′-NO_3_) (L-arg)(2,2′-bpy)]·NO_3_}_n_ complex was not an effective precursor for the electroless metallization of Nd:YAG laser activated coatings. An attempt was made to determine the influence of the precursor chemical structure on the obtained metallization effects.

## 1. Introduction

In recent years, many methods of selective electroless metallization for non-conductive polymer materials have been developed. Extensive research was aimed especially at designing new coatings containing various electroless metallization precursors, suitable for applications in electronics [1,2,3,4]. Chemical surface activation is used to the greatest extent in industrial practice. It consists of immersing the entire sample in an SnCl_2_ solution and then in PdCl_2_ in a two-stage process, or directly in a SnCl_2_/PdCl_2_ solution in a one-stage process. In the last step, the activated samples are placed in an appropriate electroless plating bath. Despite the simplicity of the electroless metallization process, it also has some disadvantages. Some of them are the lack of selective surface activation and the poor adhesive strength between the deposited copper layer and the polymer substrate. The first disadvantage is an especially significant drawback in electronic applications.

The use of lasers enables selective surface activation and subsequent electroless metallization. Laser surface activation can be performed in two main approaches. In the first approach, indirect laser modification is performed, which later requires the chemical activation of the irradiated surface [5,6,7]. Indirect activation can take place in a liquid [5] or an atmospheric [6,7] environment by modifying the surface of the polymeric material to enable the implementation of catalysts in the next stage, e.g., palladium, and thus to obtain a fully active surface [8,9]. In the direct method of surface activation, the material can be simultaneously modified and metallized. However, this requires the use of appropriate compounds named metallization precursors, added at the processing stage to the polymer material [10,11]. The precursors may be added to the total volume of the material (e.g., during the extrusion and injection molding) or be present in polymeric coatings which cover the substrate. The method of such activation is often known as laser direct structuring (LDS).In the LDS method, laser radiation causes the ablation of the material surface layer, mainly the organic part, causing the precipitation of metal on the surface, which forms active catalytic centers. LDS is an innovative technology with great potential for the production of metallized patterns on non-conductive materials with design flexibility and high precision. This method can be used to manufacture molded interconnect devices (MIDs) combining mechanical and electronic functions in one injected element.

The precursors used in the LDS method can be divided into inorganic and organometallic compounds. In recent years, research has been conducted on the application of organometallic complexes that can act as precursors in the metallization processes of polymer products. The information about palladium, copper or nickel acetates, acetylacetonates or copper complexes used as effective precursors of electroless metallization can be found in the literature [1,12,13,14,15,16,17]. Inorganic metal compounds such as copper, chromium in the form of CuO·Cr_2_O_3_ [18] oxide or hydrogen phosphate Cu_2_(OH)PO_4_] [19] were also used. Beside copper compounds, antimony-doped tin oxide [20] or multi-wall MWCNT/PP carbon nanotubes [21] were added to the polymer matrix.

In this work, a comparative assessment of Cu(acac)_2_ and {[Cu(μ-*O,O*′-NO_3_) (L-arg) (2,2′-bpy)]·NO_3_}_n_ as potential precursors for laser activated electroless metallization of polymeric materials were made. Antimony(III) oxide Sb_2_O_3_, as an additional component of the coatings was used as an absorber of infrared radiation. The main goal of the work was to determine the compounds’ abilities to be effective precursors for electroless metallization, leading to the formation of a copper layer on the surface of laser irradiated coatings.

An additional goal of the research was to determine the effect of Sb_2_O_3_, as a compound that absorbs infrared radiation very well, on the effects of surface activation [22,23]. It was assumed that the presence of Sb_2_O_3_ in the coating can increase the amount of locally precipitated copper active catalytic sites. Finally, as two precursors with various chemical structures were tested, it can facilitate a more appropriate selection of a new precursor in the future.

## 2. Materials and Methods

### 2.1. Materials

The following materials were used in the research:

- Polinitrate(V) complex of [(2-amino-5-guanidine-pentane) (mi-O,O′-nitrate(V)) (2,2’-dipyridile) copper (II)]{[Cu(μ-*O,O*′-NO3)(L-arg)(2,2′-bpy)]·NO3}n designated as compound A;
- copper (II) acetylacetonate Cu(acac)_2_ (Sigma Aldrich, Saint Louis, MO, USA), designated as compound B;
- antimony (III) oxide Sb_2_O_3_ (particle size < 250 nm) (Sigma Aldrich, Saint Louis, MO, USA);
- polyurethane resin B4060 (Haering, Bubsheim, Germany);
- polycarbonate (PC) Xantar 19 UR (DSM Engineering Plastics, Nancy van Heesewijk, Netherlands);
- autocatalytic copper metallization bath M-Copper-85 (MacDermid- Poland, Łysomice, Poland);
- formaldehyde 36%, HCHO (POCH, Gliwice, Poland), molecular weight 30.03 gmol^−1^;
- two-compound adhesive Araldite 2011 (Huntsman, Basel, Switzerland).

Complex A is not commercially available and has been synthesized in a crystalline form. More information on the compound is provided in the patent [24].

The total content of the additives used in the coating, including complex A or B and Sb_2_O_3_ was 20 wt%. The coating symbols and their composition are listed in Table 1.

The coating components were mechanically mixed with a polyurethane resin, and then evenly applied by pouring onto PC substrate. The PC samples were made by injection molding. The thickness of the obtained coatings was from 300 to 700 µm.

### 2.2. Laser Modification and Metallization

The coatings were treated with laser radiation ata wavelength of λ = 1064 nm and a maximum laser power of 20 W. The Nd:YAG TS-20W laser (Techsol, Bielsko-Biała, Poland) was used in this research. The applied power was 4 W, whereas the irradiated surface areas were scanned repeatedly 18 times, at a scanning speed of 800 mm/s. The coatings were irradiated at room temperature. The selection of laser irradiation parameters was carried out on the basis of experimental preliminary tests and visible optical effects on the surface of the coatings.

After laser irradiation, the coatings were immersed in a M-Copper 85 (MacDermid-Poland, Łysomice, Poland) electroless copper plating bath with formaldehyde as the reducing agent. The coatings were metallized for 60 min at a temperature of 48 °C. The pH of the metallization bath was 12.8.

### 2.3. Methodology

The thermal degradation of the investigated copper complexes was carried out using the Q500 thermogravimetric analyzer (TGA) (TA Instruments, New Castle, DE, USA). The tests were carried out in a nitrogen atmosphere under nitrogen flow rate of about 90 mL/min. The samples were heated at 5 °C/min over a temperature range from 30 °C to 1000 °C. In addition to the dependence of the mass change of the sample on the temperature (TG), its first derivative was also determined (DTG).

The surface topography of the coatings was examined with a scanning electron microscope (SEM) SU8010 (Hitachi, Tokyo, Japan) equipped with a dispersive X-ray detector (EDX). Prior to high-resolution surface topography analysis, a thin conductive gold layer was deposited onto the coatings in a vacuum chamber. In the case of the elemental analysis of the coatings, their surface was not coated with gold.

X-ray photoelectron spectroscopy (XPS) experiments were carried out using an R3000 spectrometer (VG Scienta, Sweden) equipped with an Al anode emitting X-ray photons with an energy of 1486.6 eV. The main purpose of this study was to detect and determine the degree of copper oxidation in the surface layer after laser irradiation. In this work, the vector fitting method was used, allowing one to distinguish Cu(0) metallic copper from the Cu_2_O oxide form, which are difficult to distinguish because their photoelectron emission bands overlap. The vector fitting method consists of determining the reference spectra of each form of copper (Cu(0), Cu_2_O, CuO and Cu (OH)_2_), and subsequent mutual overlapping and adjusting their intensity so that their superposition corresponds as closely as possible to the spectrum recorded from the tested coatings. Details of this fitting technique can be found in previous papers [25,26,27]. In the Cu2p band, with the binding energy range from 960 to 930 eV, beside peaks derived from various forms of copper, a Sb3s peak associated with the presence of Sb_2_O_3_ in the coatings was also recorded. The shape, area and position of this peak were determined from a reference measurement of Sb_2_O_3_ in powder form. In the performed analysis, the area of the Sb3s peak is proportional (“rigidly” bound) to the Sb3d_3/2_ peak. This peak is not included in the quantitative analysis, but it is necessary for the correct model of the Cu2p band and for the correct quantitative analysis of the copper elements.

**Figure 1:**
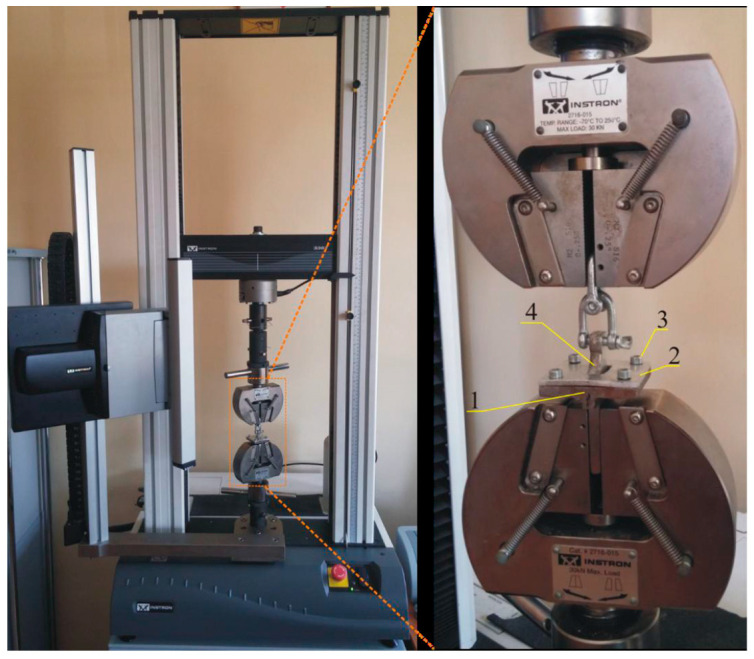
The clamps for testing the adhesive strength of coatings and deposited copper layers; the tested sample was mounted between the fixed base (1) and the cover (2) by clamping with screws (4), with a hole for a punch (4) in the cover (2).

The adhesive strength of polyurethane coatings as well as the deposited copper layer were carried out by peeling off the glued stamp. It was performed using the Instron 3367 testing machine ((Instron, Norwood, MA, USA). A metal stamp was attached to the copper layer with Araldite 2011 (Huntsman, Basel Switzerland) adhesive. After 48 h, the sample with a metal stamp was mounted in specially constructed clamps of the testing machine (Figure 1).

The measurements were carried out at a tensile speed of 2 mm/min. The adhesive strength was calculated as the maximum force per stamp surface area which adhered to the surface of polyurethane coating or copper layer. The surface area of the stamp was 130 mm^2^ (6.5 mm × 20 mm).

## 3. Results

**Figure 2:**
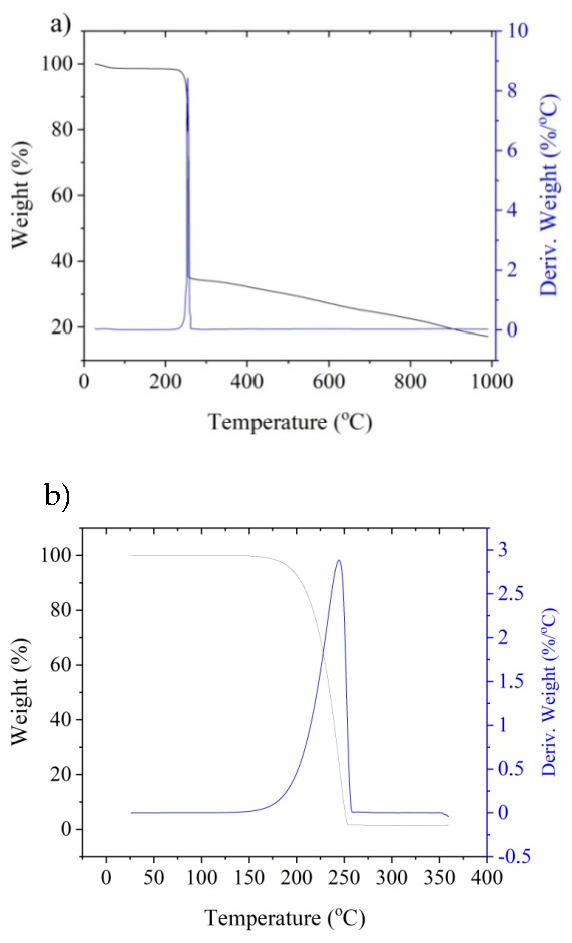
Thermogravimetric (TG) curves for: (**a**) compound A; (**b**) compound B.

Thermogravimetric analysis of the complex compounds was performed to determine their thermal degradation characteristics. This study canreflect the susceptibility of the tested compounds to thermal decomposition during laser ablation. Figure 2 shows the TG curves of compounds A and B.

The onset of weight loss for compound A begins at about 244 °C. The process of rapid weight loss lasts up to about 270 °C with the fastest weight loss at about 255 °C. In the case of compound B, the onset of weight loss occurs at about 185 °C and the mass rapidly decreases up to about 257 °C. The fastest weight loss of compound B occurs at 245 °C.

Comparing the two compounds, compound B turns out to be less thermally stable, which can result in its faster decomposition during laser irradiation and precipitation of a larger amount of metallic copper on the surface. Compound B, already at a temperature of about 260 °C, completely decomposes, while in the case of compound A, complete decomposition does not occur even at temperatures up to 1000 °C, resulting in residue within 20% of its original weight. Therefore, it can be expected that compound B can contribute to a higher ablation rate and precipitation of copper on the coating’s surface, as compared to compound A.

**Figure 3:**
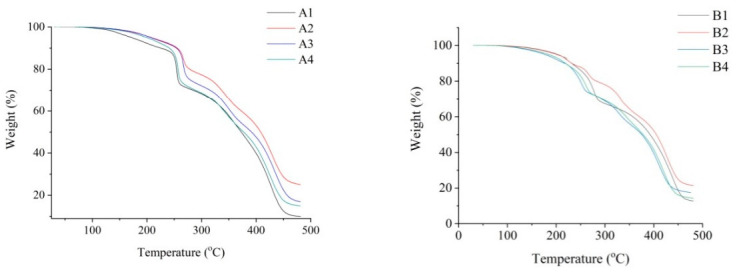
TG curves of polyurethane coatings.

Thermogravimetric analysis was also applied to coatings A and B, as shown in Figure 3.

The onset of the weight loss of coating A starts at a temperature of about 244 °Cwith the fastest weight loss at 254 °C.In the case of the coating B, the onset of weight loss occurred at about 190 °C, with the fastest weight loss at 244 °C. The total weight loss in coatings A and B was about 90% of the initial weight.

The thermogravimetric curves of the tested coatings containing complexes A and B also show that the more Sb_2_O_3_ in the coatings, the lower the total weight loss observed during the test. This is due to the fact that, of all the coating components, Sb_2_O_3_ is the one with the highest thermal resistance to degradation, as its melting point is 656 °C.

Based on the TG research, it can be assumed that due to the significant lylower degradation temperature of coatings containing compound B, the application of this compound can be a more effective way to enable electroless metallization compared to coatings with compound A. As a result of a more intensive ablation process of coatings with compound B, the amount of precipitated copper in the coating’s surface layer should be greater than in the case of coatings with compound A. A larger amount of precipitated copper should result in better effects of the metallization process, i.e., higher deposition rate and better-quality of deposited copper layers. The possibility of using TGA analysis to initially estimate the degree of degradation induced by laser radiation has been the subject of more extensive discussion in another study [28].

In order to verify the assumptions made on the basis of the TG test, the coatings containing the tested complexes were laser irradiated and subjected to the metallization process. The coatings were irradiated using a Nd:YAG laser infrared radiation with a wavelength of 1064nm. The power of the generated beam was 4W, with 18 multiplications of the scans of the coating surface with the laser beam.

**Figure 4:**
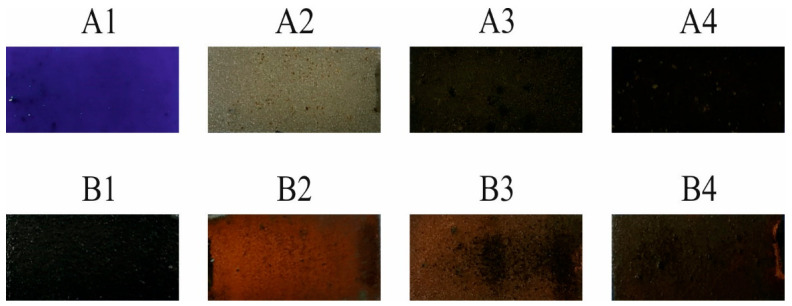
Images of irradiated and electroless metallized coatings.

The effects of the metallization process showed that if only the A or B complex compounds are present in the coating, it is not enough for the metal layer to deposit on the surface. The addition of Sb_2_O_3_ did not allow the deposition of a copper layer in all cases. In the case of coatings containing compounds A or B and Sb_2_O_3_, the copper layer was deposited only in the case of coatings with compound B. The best metallization effects were obtained for coating B2 with the highest Sb_2_O_3_ content, and with the decrease in the oxygen content in the coating, the metallizing effects deteriorated significantly (Figure 4).

As one can see, regardless of the coating’s composition containing compound A, it was not possible to deposit a copper layer. On the other hand, the copper layer was deposited on the B2 and B3 coatings. The copper layer on the B2 coating deposited on the entire laser-irradiated surface. A smaller amount of copper (visual assessment) was deposited on the B3 coating, while on the coating B4 copper was not visible without the instrumental analysis.

The preliminary visual assessment of the metallized coatings has shown that the type of the applied complex has a significant impact on the effects of the metallization process, and thus the possibility of its use as a metallization precursor. In addition, it was found that the presence of Sb_2_O_3_ had a positive effect on the metallization of coatings with compound B (the best results were obtained with the highest content of this compound).

**Figure 5:**
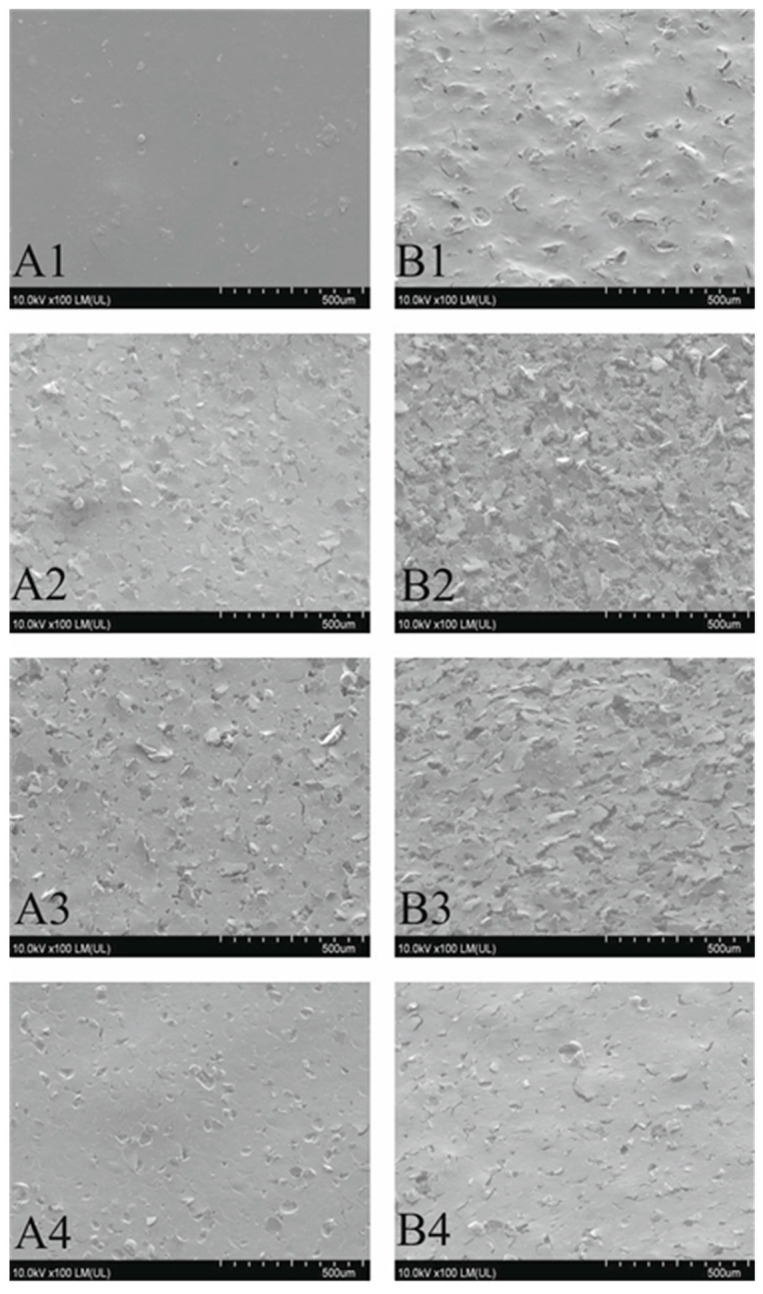
SEM images of laser irradiated coatings (coatings designation in Table 1).

To determine the cause of the positive effect of Sb_2_O_3_ on the electroless metallization results, changes in the surface structure resulting from the laser modification were investigated and analyzed. In order to illustrate the changes after laser irradiation, SEM images were taken, which are presented in Figure 5.

The smallest changes in the structure are visible on the A1 and B1 coatings. The higher the Sb_2_O_3_ content in the coatings, the more diverse is the topography of the irradiated coatings. The most changed surface can be observed for the B2 and B3 coatings, where Sb_2_O_3_ content is the highest. In addition, in the case of coatings with a high content of Sb_2_O_3_, the difference in the effects of laser irradiation between coatings with compounds A or B is clearly visible. Laser radiation caused much greater changes on the surface of coatings containing compound B. The reason for this can be the significant lylower degradation temperature of the compound B, which was determined based on TG measurements.

From the presented SEM images, it can be concluded that as a result of laser radiation, the coatings with the compound B are more prone to ablation, which results in a more changed surface morphology.

The addition of Sb_2_O_3_ to the coatings significantly changes the effects of laser irradiation manifested by the fragments of this compound present on the surface of the coating, which was confirmed by the EDX analysis. However, again, due to the lower degradation temperature of complex B, the observed changes were more intense for coatings with this complex. The much more changed surface structure of the coatings A2 and B2 compared to samples A1 and B1 is the result of the higher content of Sb_2_O_3_.

One can expect that the Sb_2_O_3_ contained in the coatings, characterized by a significant absorption of infrared radiation [23], leads to a much greater increase in the temperature of the coating surface layer and thus a much more intensive process of thermal laser ablation. An additional effect of intensified ablation process is the formation of an extremely micro-rough structure on the surface of the coatings, which may lead to greater adhesive strength of the deposited metallic layers.

The intensity of the observed changes in the surface structure decreased with the decrease in the Sb_2_O_3_ content. The surface structure of irradiated A4 and B4 coatings, i.e., samples with the coatings with the lowest Sb_2_O_3_ content, was very similar to the modified structure of coatings without this additive. The main differences in the coatings after laser irradiation with the lowest and highest Sb_2_O_3_ content are visible in the smaller number of voids formed and more exposed Sb_2_O_3_ particles. The analysis of changes in the structure of the irradiated samples showed that the presence of Sb_2_O_3_ in the coatings had a significant impact on the effects of the ablation processes.

To explain why, despite similar changes in the surface structure of irradiated coatings A and B, metallic copper layers were not obtained in the case of the first complex, photoelectron spectroscopy (XPS) studies were carried out to determine changes in the chemical structure of irradiated coatings. In the XPS method, photoelectrons are emitted from the surface layer with a maximum thickness of about 10 nm [29]. This is important information as only the outer nanoscopic layer of the modified coatings is analyzed.

Table 2 presents the quantitative results of XPS of the irradiated coatings, which lists the elements present in the surface layer of the tested coatings. It has been found that in the coatings with compound B (coatings B2, B3 and B4) the O/C values were lower as compared to coatings with compound A (coatings A2, A3 and A4).

Significant differences in the content of copper atoms in the tested coatings can also be noticed. The coatings containing compound B have a much higher copper content than the coatings with compound A. The difference may result from the chemical structure of the copper complexes and most probably relates to the amount (ratio) of copper atoms to total molecular weight of complex. The percentage of copper in complex A is less than 10%, while in complex B it is over 24%. Such a difference in the content of copper could have an impact on the amount of precipitated copper after laser irradiation coatings. It can be assumed that the higher content of copper in the surface layer of the B complex coatings results in better metallization effects.

**Figure 6:**
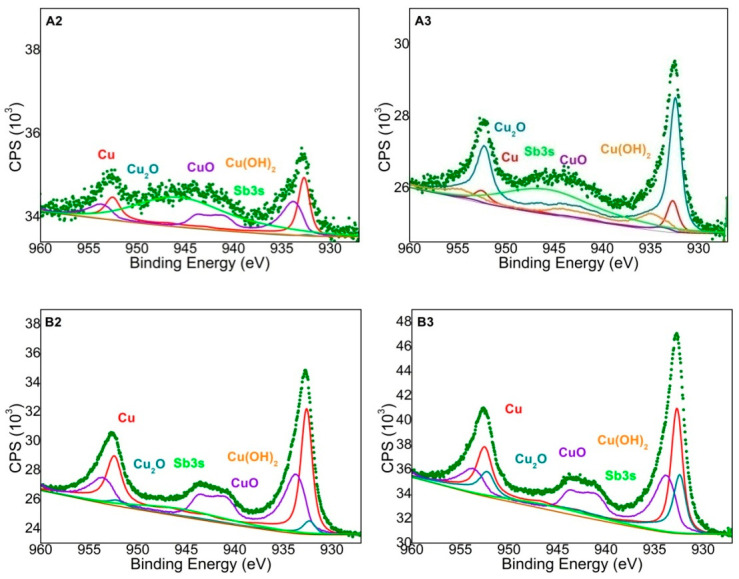
Spectra of A2, A3, B2 and B3 coatings with model spectra for Cu (0), CuO, Cu_2_O and Cu(OH)_2_ adjusted to the recorded spectra.

The XPS technique also determined the forms of copper precipitated on the surface of the coatings after laser irradiation (Table 3, Figure 6). It can be concluded that the amount of copper precipitated after laser irradiation had the crucial influence on the metallization effects.

Despite the significantly higher content of copper atoms in the surface layer of coating B3, the metallization effects of this coating were definitely worse than in coating B2. The crucial influence on the effects of metallization had probably the form in which the copper was present in the surface layer of the material, as well as the amount of the Sb_2_O_3_, which was responsible for absorbing infrared radiation. The largest amount of copper in metallic form was precipitated on the surface of the coating B2. In coatings with B compound, more than 50% of the precipitated copper was in the form of Cu (I) and Cu (II), which most likely improved the effects of electroless metallization (Table 3).

**Figure 7:**
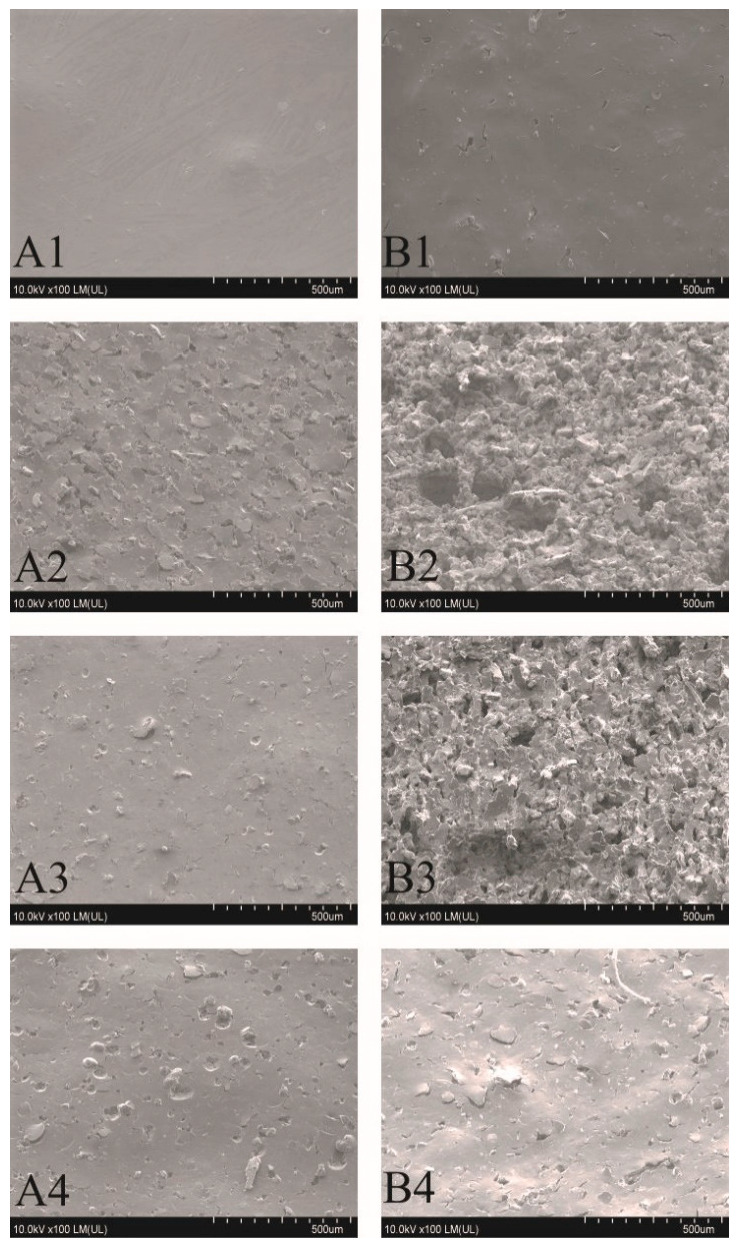
SEM images of coatings after electroless metallization.

The surface of coatings after the electroless metallization is similar to the surface structure of the coatings after laser irradiation (Figure 7).

**Figure 8:**
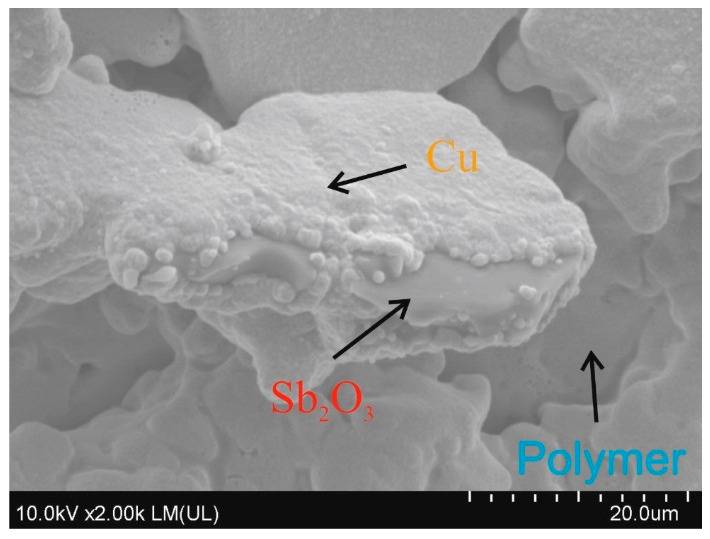
SEM image of B2 coating after electroless metallization.

On the coatings where the copper layer has been obtained, the surface structure of the deposited layer is strongly developed, and to some extent reflects the structure obtained after laser irradiation. The Sb_2_O_3_ particles exposed as a result of laser irradiation after the metallization process were covered with a copper layer (B2 and B3 coatings) (Figure 8). In the case of samples where the electroless metallization process was not successful, the surface structure reflected the structure after the laser irradiation process.

The irradiated and metallized B2 coating (Figure 7 and Figure 8) was practically completely covered with a copper layer, except for the characteristic depressions where the matrix of the coating was not subjected to thermal ablation. The EDX analysis of metallized coatings showed 46.5 at% and 13 at% of copper on the B2 and B3 coating, respectively. On other coatings, including those with complex A, no copper was detected with this technique after the metallization process.

In the previous work [15], similar studies were carried out using complex B, however, the surface modification was performer with an ArF excimer laser generating UV radiation (λ = 193nm). The surface obtained as a result of irradiation was characterized by a completely different topography than in the case of the samples presented in this article. After the coatings were irradiated with the ArF excimer laser, their surface had a conical structure, where copper precipitation occurred at the tips of the cones as a result of the degradation of the complex. Copper was also present in the lower parts of cones but in a much smaller amount. After metallization, the surface structure of the deposited copper layer mirrored the surface structure of the substrate after laser activation. The entire surface of the cones was covered with a layer of copper, but the tops of the cones were rounded, due to a significantly large amount of deposited copper. The same effects were obtained when using another copper (II) L-tyrosine complex [17]. Thus, the effects of laser modification and electroless metallization were definitely different from those presented in this work, where copper was mainly precipitated on Sb_2_O_3_ particles.

**Figure 9:**
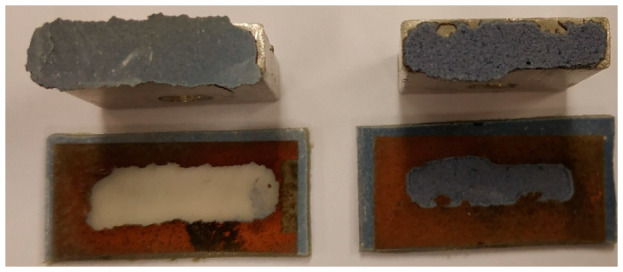
Sample photos of metallized coatings with compound B and stamps after the pull off test.

In order to check the adhesive strength of the deposited copper layers, the adhesive strength tests were carried out using tensile machine by pulling off the glued stamps. The copper layer did not peel from the polymer coating in any of the samples, because the cracking of the adhesive joint occurred at the substrate interface (polycarbonate/coating)or inside the polyurethane coating (cohesive breakout) (Figure 9). Therefore, based on the results, it can only be stated that the adhesive strength of deposited copper to coatings is greater than that values obtained in the research tests.

The adhesive strength of the polyurethane coating formed on the PC substrate was approximately 2.0 ± 0.3 MPa. The adhesive strength of the deposited copper layer, taking into account the visual evaluation of the joints after the test described above, should be greater. It is assumed that such a high adhesive strength of the copper layer is due to the specific structure formed under the influence of laser irradiation, characterized by a large surface area development caused by ablation of the matrix and irregularly shaped Sb_2_O_3_ particles. The developed surface area of the laser irradiated coatings enables the very good anchoring of the copper layer. In addition, a very rough copper layer provides a large contact area with the adhesive through which the stamp was attached, which prevented cracking of the adhesive joint at the interface between the copper layer and the adhesive.

## 4. Conclusions

The paper presents a comparison of two complex compounds as potential precursors of electroless metallization. The influence of the addition of Sb_2_O_3_ on the laser activation of the surface of the coatings containing the tested complexes was also assessed. Laser activation was performed using a Nd:YAG laser. Based on the conducted experimental studies, it was found that the complex B turned out to be an effective precursor of electroless metallization. The study also showed that adding Sb_2_O_3_ to the coatings resulted in greater changes in the surface topography of the coatings under the influence of infrared laser radiation.SEM images showed that in the irradiated areas, a rough surface structure was formed, especially on the coatings B2 and B3 as well as A2 and A3. Additionally, copper was precipitated on the irradiated coatings B2 and B3, which acted as active catalytic centers for electroless metallization. On the laser-activated surfaces of the coatings with complex B, the protruding fragments of the coatings were Sb_2_O_3_ particles on which copper had been precipitated. It was proved using the XPS technique that after laser irradiation of coatings with compound B, copper in the form of Cu, CuO, Cu_2_O and to a small extent in the form of Cu(OH)_2_ was precipitated. Most likely, in the coatingB2, the form in which the copper was present in the surface layer of the material had a crucial influence on the metallization effects. The addition of Sb_2_O_3_, which was responsible for absorbing infrared radiation, led to better metallization effects. The obtained copper layers (on B2 and B3 coatings) were characterized by a very good adhesive strength, exceeding 2MPa (coating and substrate adhesive strength).

## Figures and Tables

**Table 1:** Coatings compositions and designation of coatings.

Coating Symbol	The Content of Compounds in the Coating: (wt%)		
	Complex A	Complex B	Sb_2_O_3_
A1	20	–	–
A2	10	–	10
A3	15	–	5
A4	16.67	–	3.33
B1	–	20	–
B2	–	10	10
B3	–	15	5
B4	–	16.67	3.33

**Table 2:** Content of copper, oxygen, carbon and antimony in coatings.

	Cu (at%)	O (at%)	C (at%)	Sb (at%)	O/C
A1	0.19	29.86	69.97	0.00	0.43
A2	1.15	39.47	52.08	7.29	0.76
A3	1.96	38.12	54.66	5.25	0.70
A4	1.84	31.30	62.37	4.49	0.50
B1	6.69	28.00	65.30	0.00	0.43
B2	4.37	31.98	59.74	3.91	0.54
B3	7.18	29.41	60.06	3.34	0.49
B4	7.10	29.10	61.88	1.92	0.47

**Table 3:** Percentage share of various forms of copper in irradiated coatings.

Coating	Cu (0) (%) (EB = 932.7 eV)	CuO (%) (EB = 933.9 eV)	Cu_2_O (%) (EB = 932.4 eV)	Cu(OH)_2_ (%) (EB = 935.0 eV)
A2	40.87	56.52	0.87	1.74
A3	11.73	3.57	63.78	20.92
B2	44.39	49.20	6.18	0.23
B3	37.19	39.97	22.84	0.00

## Data Availability

Data sharing not applicable.
